# Central Auditory Nervous System Stimulation through the Cochlear Implant Use and Its Behavioral Impacts: A Longitudinal Study of Case Series

**DOI:** 10.1155/2021/8888450

**Published:** 2021-04-27

**Authors:** Marina Isabel Cavalcanti, Liliane Aparecida Fagundes Silva, Maria Valéria Schmidt Goffi Gomez, Tsuji Robinson Koji, Ricardo Ferreira Bento, Ana Cláudia Martinho de Carvalho, Matas Carla Gentile

**Affiliations:** ^1^Department of Physiotherapy Speech and Language Pathology and Audiology and Occupational Therapy, School of Medicine, University of Sao Paulo (USP), Sao Paulo, Brazil; ^2^Cochlear Implant Group of the Clinical Hospital, School of Medicine, University of Sao Paulo, Sao Paulo, Brazil

## Abstract

The purpose of this study was to investigate, over a period of five years, the cortical maturation of the central auditory pathways and its impacts on the auditory and oral language development of children with effective use and without effective use of a Cochlear Implant (CI). A case series study was conducted with seven children who were CI users and seven children with normal hearing, with age- and gender-matched to CI users. The assessment was performed by long-latency auditory evoked potentials and auditory and oral language behavioral protocols. The results pronounced P1 latency decrease in all CI users in the first nine months. Over five years, five children with effective CI use presented decrease or stabilization of P1 latency and a gradual development of auditory and oral language skills, although, for most of the children, the electrophysiological and behavior results remained poor than their hearing peers' results. Two children who stopped the effective use of CI after the first year of activation had worsened auditory and oral language behavioral skills and presented increased P1 latency. A negative correlation was observed between behavioral measures and the P1 latency, the P1 component being considered an important clinical resource capable of measuring the cortical maturation and the behavioral evolution.

## 1. Introduction

In a healthy central nervous system, the auditory pathway and cortical area organization occurs through acoustic stimulation received in the first years of life, and it enables children to develop oral language skills. This organizational process occurs through the neuroplasticity phenomenon [1].

In children with severe to profound sensorineural hearing loss (SNHL) acquired before the oral language development, the literature indicates impairment in the child's general development and significant restriction in oral language development, resulting in delays in biopsychosocial sphere and learning acquisition and development, directly impacting their quality of life [2, 3].

Such impairments are manifested according to the degree, type, location, and duration of the loss, as well as the etiology of the hearing loss, the age of diagnosis, and auditory intervention. Children affected by hearing loss exhibit impairment in the perception of acoustic cues fundamental to the comprehension of speech, which has a negative impact on the natural learning of oral language [4].

In the brain, the sensorial deprivation resulting from SNHL causes a drastic decrease in spiral ganglion neurons and demyelination of the residual ganglion neurons, thus promoting a decrease in the activity of the central auditory nervous system (CANS) [5].

CI stands out as an important technological resource that allows individuals with SNHL to experience acoustic stimuli through direct electrical stimulation of the cochlear nerve. The electronic device is surgically inserted into the cochlea, and the electrical stimuli produced are conducted through the auditory pathway to the cortex in the brain, where the electrical information is interpreted [6]. During childhood, the CANS present maximum neuroplasticity, the critical period, and the exposure to acoustic stimuli enables the reorganization of the system, enabling the hearing and linguistic capabilities to be acquired [7–11].

It is known that the gradual development of auditory skills, both in CI users and in normal-hearing children, is associated with cognitive processes such as attention, memory, and auditory experiences acquired [12]. Oral language development occurs simultaneously with auditory function maturation and is accompanied by other capabilities associated with development in children [13]. Therefore, longitudinal monitoring in children using CIs is necessary for the extent that the development of auditory and oral language capabilities occurs through a gradual and interdependent process.

Currently, there are various specific protocols capable of providing information on the development of auditory and oral language capabilities after CI surgical intervention [11, 14–16]. The measurement of long-latency auditory evoked potentials (LLAEPs) has been a widely used clinical resource because it enables the verification of the integrity and maturation of the CANS pathways after CI use. Studies have pointed out that the stimulation promoted by the CI makes SNAC maturation possible; such transformations are capable of promoting the gradual auditory and oral language skills development. In such a way, progress in behavioral performance of auditory and oral language skills has been correlated with a decrease in P1 component latency [17, 18].

Considering that SNAC maturation occurs over time and auditory and oral language skill development is part of a process that occurs gradually over sensory stimulation by CI use, the importance of longitudinal monitoring is emphasized, for a long period of time, highlighting the importance of the present study. Thus, this study is expected to contribute to the understanding of the SNAC functioning and maturation and the benefits of this technology.

Thus, this research aimed to investigate, over a period of five years, the cortical maturation of the central auditory pathways and its impacts in the auditory and oral language development of children with effective use and without effective use of a Cochlear Implant (CI).

## 2. Case Series

This is a prospective longitudinal case series study, approved by the Research Ethics Committee under number 0319/11, performed in two groups: a study group (SG), composed of children who received surgical indication for CI, and a control group (CG), composed of normal-hearing children.

The SG sample was composed of seven children, with a mean age of three years and three months at the time of activation, a mean time of sensory deprivation equal to one year and six months, and a mean time of hearing aid (HA) use prior to the CI equal to one year and nine months (Table 1). These children presented bilateral, congenital, severe, and/or profound SNHL, used bimodal stimulation, attended speech-language-hearing therapy (at least once a week), and did not present neurologic and cognitive impairment or other alterations that might affect auditory or language development. Each SG child was assessed at four different moments: prior to CI activation and after three months, nine months, and five years of CI use.

The CG was composed of seven children with chronological age- and sex-matches to the SG; thus, the average age of these children was eight years and two months (minimum of six years and maximum of nine years). These subjects presented normal pure-tone threshold audiometry (better than 20 dB HL in any of the evaluated conventional frequencies [4]), normal speech recognition threshold (up to 10 dB HL above tritonal average) and speech recognition percentage (index superior or equal to 88% [4]), normal acoustic immittance measures, with type A tympanometric curve, the and presence of ipsilateral and contralateral acoustic reflexes [19]. Moreover, they presented the absence of neurologic, cognitive, or language impairments (who were dismissed through interviews conducted with the parents) and had not previously attended speech-language-hearing therapy. The CG was submitted to only one assessment to compare the results of the SG children after five years of CI use with their normal hearing peers of the same chronological age.

Before the start of the research procedures, the researcher checked that the CI was functioning properly through the detection of speech and ambient sounds. In addition, IC users underwent periodic follow-up at the cochlear implant group, where they conduct tests and map the electronic device.

The LLAEP assessment was performed in a sound-attenuated room, with the child in an alert state, sitting comfortably in a reclining chair. They were instructed to remain as quiet as possible during the procedure and watch a cartoon without sound. The equipment used was a Smart EP USB Jr Intelligent Hearing System (IHS 5020). For the positioning of the electrodes, the International Electrode System IES 10–20 [20] was considered to be fixed. After the skin was cleaned, with conductive paste for electroencephalography, the reference electrodes (negative) were positioned on A1 and A2 (lobes of the left and right ears, respectively), the electrode ground was positioned on Fpz, and the active electrode was positioned on Cz. Electrode impedance levels were maintained below 3 kOhm.

The stimuli were presented in a sound-field system with loudspeakers positioned at an azimuth of 90°, 40 cm away from the implanted side, with the speech stimulus, syllable/ba/, presented with an interstimulus interval of 418 ms, with the sound level set at 70 dBnHL, with a presentation rate of 1.9 stimulus per second. Furthermore, the high- and low-pass filters were set between 1 and 30 Hz with a gain of 100,000 and a response analysis window of 0 to 500 ms.

The criterion for identification and analysis of the P1 component was based on the literature [21, 22], the first positive peak of greater amplitude being measured, with latency values between 50 and 300 ms that presented reproducibility [10].

For auditory and language behavior skills, two parent-perception auditory and language behavioral measure protocols were applied at all assessment moments: the Meaningful-Auditory-Integration-Scale- (IT-MAIS/MAIS-) adapted questionnaire and the Oral Language Assessment Questionnaire, adapted from the Meaningful Use of Speech Scales (MUSS).

Both questionnaires were applied as interviews with the parents, composed of 10 simple questions related to the child's auditory (IT-MAIS/MAIS) and linguistic (MUSS) behavior in different day-to-day situations. The answers given by the parents/guardians were scored from zero to four points (0 = never; 1 = rarely; 2 = occasionally; 3 = frequently; and 4 = always). In the end, the total number of accumulated points was summed, which could reach a maximum score of 40 points; the totals were then converted into percentages [14, 23–25].

After nine months of CI use, the children were evaluated using the speech perception assessment through the Glendonald Auditory Screening Procedure (GASP) [26] and the Speech Sound Perception Assessment Protocol for Children with Hearing Loss-Word List [16].

Both tests were applied in a sound booth in a sound-field system using a clinical audiometer, with stimuli presented at an intensity of 60 dB SPL. The loudspeakers were positioned at an azimuth of 45°, and to make it impossible for orofacial reading, the evaluator used a shield beneath the eyes while this procedure was performed. Before each test, the children were verbally instructed with permission to orofacial reading.

The GASP test is composed of six components that evaluate speech perception of children with severe and/or profound hearing loss. The tests were conducted sequentially, according to the auditory performance of each child [26]. Children were classified according to their performance in accordance with their auditory skills: detection, discrimination, recognition, and comprehension.

The Speech Sound Perception Assessment Protocol for Children with Hearing Loss-Word List is composed of 20 disyllable Brazilian Portuguese words [16]. The list of words was presented by voice, with no repetitions or pauses between the phonemes. The answers were computed in percentages, summarizing the total words and phonemes correctly produced. Considering that, for the child to perform this test, they needed to have auditory open set recognition capabilities, this protocol was used only in the last assessment.

The Wilcoxon test was used to compare the results of the P1 latency, as well as those obtained in the IT-MAIS/MAIS and MUSS protocols throughout the follow-up. Moreover, to correlate the results of the LLAEP assessments and the auditory and oral language behavioral measures, the Spearman correlation test was used. The level of significance adopted was 5%.

## 3. Results

**Figure 1:**
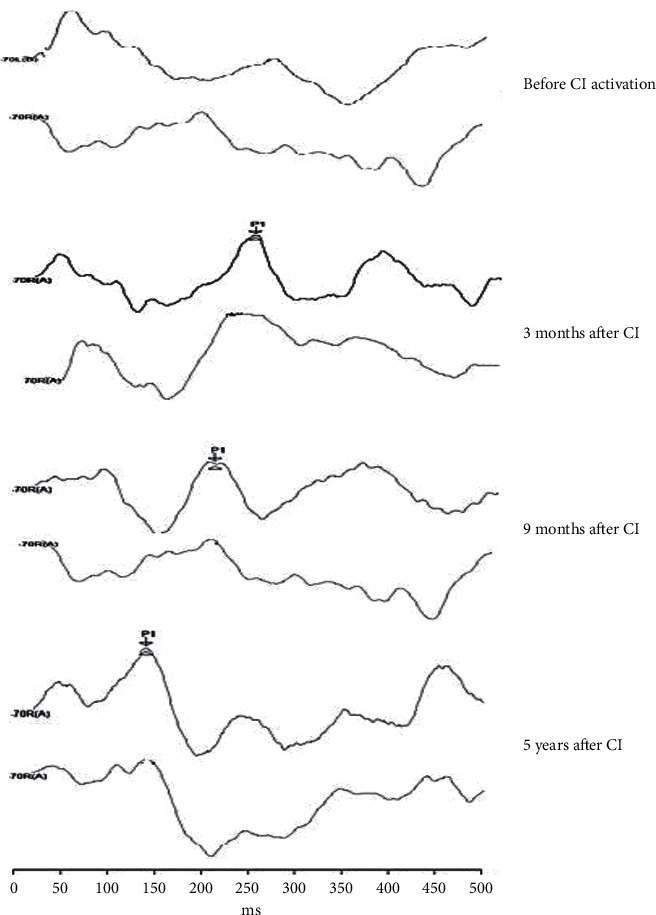
LLAEP trace recording of a child CI user.

**Figure 2:**
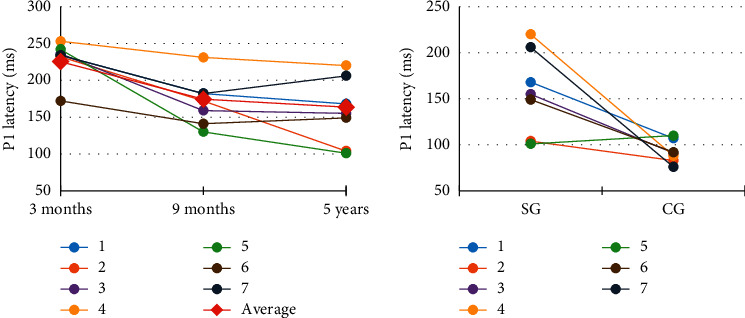
Individual SG profiles of progression of the P1 latency and comparison of results obtained between SG and CG in the last evaluation.

CANS maturation could be observed throughout the five-year period of CI use, being that, at the moment prior to the CI, the absence of electrophysiological response was observed in all the children, but after of three months of CI use, all individuals presented the P1 component (Figure 1). A P1 latency decrease over time was observed in all of the children evaluated from the third month to the ninth month of CI use; from the ninth month of use up to five years, a gradual decrease or stabilization of P1 latency was observed. However, when the P1 latency of the SG children, after five years of CI use, was compared to that of the CG, it was observed that most of the SG children still presented longer latency than their normal hearing peers (CG) (Figure 2).

Two children (6 and 7) presented increase in P1 latency after five years of CI use. These two children stopped the effective use of the CI after nine months; therefore, they were excluded from all the comparison analyses between nine months and five years of CI use; so, the subsequent analysis was only of the evolution of the children who use effectively the CI. When comparing the longitudinal results of the P1 component, a tendency toward statistical significance was observed between each of the assessments (*p* value = 0.0625).

**Figure 3:**
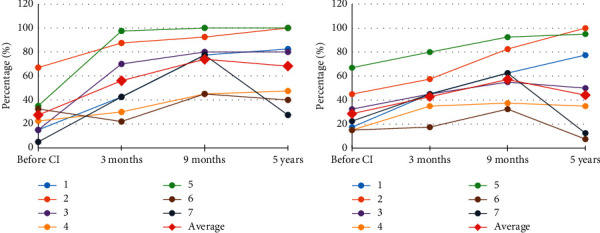
Individual profiles of progression of IT-MAIS/MAIS and MUSS results.

Regarding the IT-MAIS/MAIS protocol, evolution of the auditory skills in the parents' perception throughout the time of CI use was noted in most of the children; in this sense, one of the children reached the maximum value nine months after CI use. On the other hand, it was noted that those two aforementioned children (6 and 7) showed worsened results after five years of using the electronic device. For the MUSS protocol measures, evolution was observed in the communicative capabilities in the parents' perception throughout the time of CI use in most of the children evaluated. Nevertheless, three children were noted to obtain lower scores after approximately five years of CI use (Figure 3).

In the comparison analysis through time, in both IT-MAIS/MAIS and MUSS protocols, a significant difference was observed between the results obtained before and after three months of CI use and between three and nine months of CI use (Table 2).

Evolution was also observed in the auditory skills in most of the children evaluated through the performance on the GASP. However, three children had not acquired skills beyond the detection level even after five years of CI use (Table 3).

**Figure 4:**
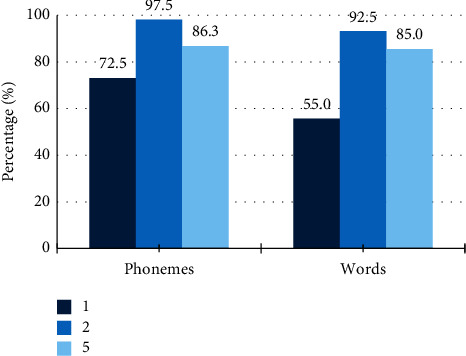
Percentage of phonemes and words correctly repeated on the words repetition test.

Concerning speech perception through the word repetition test, it was possible to obtain data from three children, considering that, for this assessment to be conducted, open set word recognition skills are necessary (Figure 4).

**Figure 5:**
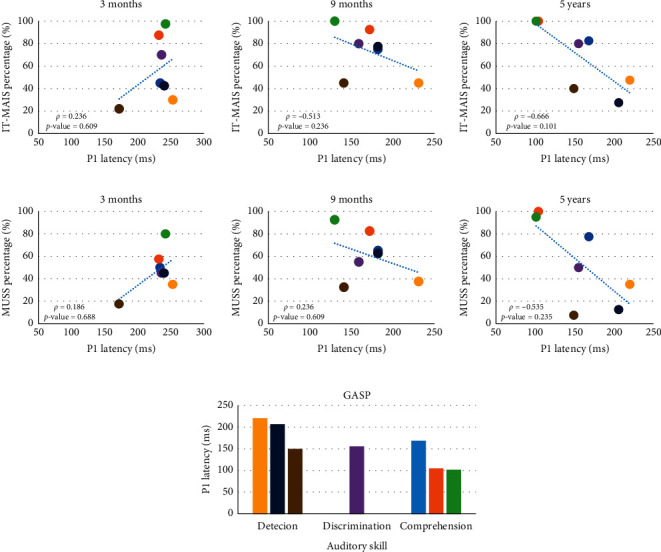
Correlation between the auditory and language behavioral measures and P1 component latency.

When analyzing the correlation between electrophysiological and behavioral measures, no statistically significant correlation was observed (*p* value >0.05). Nonetheless, after nine months of CI use, there was a tendency toward a negative correlation between behavioral measures and P1 latency, with the best results on the behavioral tests corresponding to the lowest latency values. Concerning the GASP, after nine months of CI use, all the children had detection skills. After five years of CI use, shorter latency was obtained in the children with better auditory skills (Figure 5).

It was not possible to perform correlation analysis between the latency of the P1 component and the Word List results, since the number of children who performed this assessment was small.

## 4. Discussion

The purpose of the present study was to investigate the cortical maturation of the central auditory pathways and its impacts in the auditory and oral language development, over a period of five years, of children with effective use and without effective use of a Cochlear Implant (CI).

By CANS stimulation through CI, gradual changes in the morphology and functionality of the auditory cortex can be observed, promoting additional neuronal connections, increase in the number of neurons that become responsive to sound stimuli, expanded dendritic branching, increased neuronal myelination, and improved synaptic connections and synchronization. Thus, these anatomical and physiological changes result in a gradual increase in the transmission speed of the nervous impulse due to neuroplasticity, and consequently, changes in the latency and morphology of LLAEP components can be observed in the long term [4, 6, 9, 27].

The P1 is the component most frequently observed in young children, being observed around 100–150 ms, which decreases as the chronological age increases, and the N2 component is the second most commonly observed component in children, being observed around 200–250 ms. The components N1 and P2 appear later in the maturation process and are originated from a bifurcation between the components P1 and N2 [28–30].

They are all part of the exogenous electrophysiological response to the auditory system, and the P1 and N1 components reflect of the auditory pathway integrity, neural encoding, perception and detection of acoustic stimuli, discrimination, and temporal processing, and the P2 and N2 components are related to the processing of classification, decoding of acoustic stimuli, discrimination, and temporal processing [30–33].

The results from this study revealed a faster decrease in P1 latency during the first nine months of auditory experience. Over the five-year study period, children who continued with effective CI use presented decrease or stabilization of P1 latency, which corroborates other literature findings [34–38]. However, the greatest decreases in P1 latency occurred in the first nine months of CI use, demonstrating that this period is crucial in auditory development, as it allows quick transformations in the CANS.

The literature supports that these transformations occur more sharply in the first months after CIs are activated, as the latency of the P1 component of children using CIs comes closer to that of their normal hearing peers after approximately eight months to two years of use [27, 36–39].

After approximately five years of CI use, it was noted that P1 latency was still longer than the expected chronological age in most of the children.

It is known that various intrinsic and extrinsic factors influence CANS development, and these factors could justify the heterogeneous results obtained in this population. In this study, after an average of five years of CI use, two children presented an increase in P1 latency, as well as worsening hearing and language performance assessed from the parents' perspective. It is important to highlight that these two children stopped effective CI use, which would justify this finding. Effective CI use is known to be an indispensable factor in CANS development, since the lack of auditory stimulation causes the reduction of neuronal activity in the CANS [6, 22].

Most of the children who used the CI effectively progressively evolved regarding auditory performance and oral language skills, both in the parents' perception and in the speech perception tests. Studies have demonstrated that the transformations in the CANS promoted by the effective use of the CI make it possible to develop gradual auditory and oral language skill development [39–44].

In this research, it was observed that, after nine months of CI use, all the children presented detection skills; after five years of CI use, three of them attained maximum auditory skills (comprehension), providing correct answers for more than 50% of the listened words. A relevant study described the existence of a gradual pattern in the development of the auditory skills of children using CIs; after nine months, the children are expected to be capable of detecting sounds and to start identifying words, and after five years, they are expected to recognize words in an open set, i.e., they are capable of recognizing and extracting a word's phonemic information only by listening to it, having, therefore, developed the listening comprehension skill [41].

The children who discontinued effective CI use presented a sharp decrease in auditory and oral language performance. It is important to highlight that, up to the ninth month of CI use, these children consistently used the CI, with orality as their main means of communication. Nevertheless, after five years of CI use, they had established Brazilian Sign Language as their main means of communication, which justifies the decrease in the hearing and language protocols' score.

Studies suggest that the development of auditory and oral language skills in the population of children using CIs can be close to the language profile of normal-hearing children whose chronological age is equal to the time of CI use and that it is expected that, after five years of use of the device, the children will have reached the maximum auditory and language performance [41, 42].

In this study, only two children achieved the maximum auditory performance, and one achieved the maximum performance of oral language. However, these findings corroborate other studies that demonstrated that not all children using CIs manage to match their normal-hearing peers' performance and/or meet their relatives' expectations [43, 45–51]. This happens because of countless factors that can have an impact on auditory and oral language development, such as etiology, CANS integrity, time of sensorial deprivation, age at surgery, consistent CI use, postsurgical follow-up, family participation, and others [18, 42].

When correlating the electrophysiological and behavioral measures, although the significance value adopted in this study was not reached, the findings demonstrated that there was an inverse proportion between the P1 latency values and the improvement of the auditory and linguistic skills, so that the children who presented shorter latency also presented better behavioral performance. The small sample number probably made the correlation impossible between the physiological and behavioral measures of auditory and language skills with statistical significance. A previous study, which compared the results of the latency values of the P1 component with the IT-MAIS questionnaire results in a group of 11 children using CIs, observed a negative correlation between these variables, demonstrating that shorter latency values were observed in children with better behavioral results [51].

Even so, the present study corroborates the evidence in the literature that has shown the proportional development between P1 latency and several behavioral assessment protocols, and for this reason, the P1 component has been highlighted as an important biomarker capable of quantitatively measuring the maturation of the SNAC and predicting the behavioral performance of auditory and oral language skills [38, 42, 52–56].

Finally, it is important to highlight the difficulty found in guaranteeing a large number of samples in studies of longitudinal magnitude, especially considering the clinical and demographic characteristics of this specific population, residing in a country with continental dimensions. Studies with larger populations are necessary to confirm and strengthen the results of this study.

## Figures and Tables

**Table 1:** Sample characterization.

Subject	Gender	Etiology	CI side	Time of sensorial deprivation (months)	Time of hearing aid use prior to CI (months)	Age at activation (months)	Effective use of the CI	CI maker
1	M	Compound heterozygous in the CDH23 gene	L	6	12	20	Yes	Med-El
2	F	Mutation c.35delG in the GJB2 gene in homozygous	R	9	21	31	Yes	Neurelec
3	F	Genetic to clarify	R	42	24	66	Yes	Med-El
4	M	Genetic to clarify	R	29	6	35	Yes	Med-El
5	F	Idiopathic	R	24	24	49	Yes	Neurelec
6	F	Genetic to clarify	R	12	9	21	No	Cochlear Corporation
7	M	Usher syndrome	R	18	36	54	No	Neurelec

**Table 2:** Comparison of the IT-MAIS/MAIS and MUSS results between the different moments of assessment in the children using CI.

Protocol	Time of assessment	Average	Standard deviation	V-test	*p* value^+^
IT-MAIS/MAIS	Prior to CI	27.42	20.35	2	0.0468^*∗*^
	3 months	56.00	29.17		
				0	0.0222^*∗*^
	9 months	73.92	21.45		
				0	0.1814
	5 years	82.00	21.46		

MUSS	Prior to CI	30.64	17.96	0	0.0222^*∗*^
	3 months	46.42	17.82		
				0	0.0222^*∗*^
	9 months	66.00	19.59		
				4.5	0.4982
	5 years	71.50	25.27		

^#^ *p* value with statistical difference, ^+^*p* value by the Wilcoxon test.

**Table 3:** Result of the auditory skills obtained through the GASP protocol.

Performance in the GASP protocol		
Subjects	9 months	5 years
1	Detection	Comprehension
2	Detection	Comprehension
3	Detection	Discrimination
4	Detection	Detection
5	Detection	Comprehension
6	Detection	Detection
7	Detection	Detection

## Data Availability

The data that support the findings of this study are available on request from the corresponding author. The data are not publicly available due to privacy or ethical restrictions.
